# Bioavailability of Oil-Based and **β**-Lactoglobulin-Complexed Vitamin A in a Rat Model

**DOI:** 10.5402/2013/270580

**Published:** 2013-02-19

**Authors:** Ying Liu, Ju-Jean Shaw, Harold E. Swaisgood, Jonathan C. Allen

**Affiliations:** Interdepartmental Program in Nutrition, Department of Food Bioprocessing and Nutrition Sciences, North Carolina State University, Raleigh, NC 27695, USA

## Abstract

**β**-Lactoglobulin is capable of binding fat-soluble compounds including vitamin A palmitate and is suggested to specifically enhance intestinal uptake of retinol. In this study, bioavailability of a vitamin-A-retinyl palmitate complex in skim milk and in water-based liquids was investigated in vitamin-A-depleted rats. First, rats were fed a vitamin-A-free pellet diet for 6 wk and were thereafter gavage-fed with vitamin A in oil, vitamin-A-**β**-lactoglobulin complex, vitamin A in oil + skim milk, and vitamin-A-**β**-lactoglobulin + skim milk for 2 wk and 42 wk. Vitamin A repletion, as judged by vitamin A accumulation in serum and liver, occurred in all the treatments. Vitamin-A-**β**-lactoglobulin complex treatments had statistical equivalence with oil-based vitamin A treatments. In a second experiment, vitamin-A-depleted rats were fed UHT-processed skim milk fortified with either oil-based or freeze-dried **β**-lactoglobulin-complexed retinyl palmitate. Liver and serum vitamin A were analyzed by HPLC to indicate vitamin A status in the rats. Results showed no significant difference in bioavailability of retinyl palmitate from milk made with either regular oil-based or **β**-lactoglobulin-complexed fortifiers. The vitamin-A-**β**-lactoglobulin complex, being water soluble, may be useful for fortification of nonfat products.

## 1. Introduction

Vitamin A has long been known to be crucial to normal vision and control of differentiation of epithelial cells in the digestive tract, respiratory system, skin, and bone [1]. It is also important in cell replication, growth, and maturation of the nervous and immune systems. Because of the important role of vitamin A, fluid milk products have been fortified with vitamin A (along with vitamin D) since the 1930s to reduce the incidence of disorders caused by vitamin deficiency in the USA. Vitamin A addition to whole milk is optional, but low fat and nonfat milk must be fortified with vitamin A so that each quart contains >2000 IU [2]. Vitamin-A-fortified skim milk is either prepared with oil-based carriers or water-based emulsions. Currently, the acceptable deviation range of vitamin A is 100% to 150% of the FDA-specified concentration [3]. 

However, nonfat or low-fat milk products often do not comply with nutrition labeling requirements over their entire shelf life. This may be due to analytical methods and loss of vitamin during processing and storage [4, 5]. Furthermore, the degradation of vitamin A generally parallels the oxidative degradation of unsaturated lipids.

*β*-Lactoglobulin is a major whey protein (7 to 12% of skim milk total proteins) found in bovine milk [6]. It has the ability to bind in vitro certain hydrophobic molecules such as retinol and fatty acids [7]. *β*-Lactoglobulin shares similar amino acid sequence and tertiary structure with retinol-binding protein (RBP), the main protein involved in the transport of retinol in serum. Retinol binds with considerable affinity to *β*-lactoglobulin in a molar ratio of 1 : 1 [8, 9]. Retinyl palmitate appears to bind in both the *β*-barrel cavity and in an external *α*-helix pocket, with the palmitate moiety binding on the external hydrophobic pocket between the *α*-helix and the *β*-barrel. *β*-Lactoglobulin was capable of binding 2 mol of retinyl palmitate per mol of protein [10]. Retinol binding at the external site was not evident in crystallographic studies [11]. Wang and his colleagues suggested [10] that *β*-lactoglobulin should be an excellent carrier of retinyl palmitate for use as an additive to milk and other foods, even if this is not the evolutionary function for this protein [12]. The purpose of this study was to test the bioavailability of vitamin A from this carrier.

## 2. Materials and Methods

### 2.1. Experimental Design

#### 2.1.1. Experiment 1

Male Wistar rats (Charles River Breeding Laboratories, Raleigh, NC, USA), 23 d old, were randomly housed (16 groups of 3 rats) 3 per cage for 3 wk and then in individual cages for another 3 wk. The animals had free access to water and AIN-93G pelleted diet (positive control diet groups contained 4000 IU vitamin A/kg) or a vitamin-A-free AIN-93G pelleted diet (deficiency groups) for 3, 5, or 6 wk. AIN-93G pelleted and vitamin, A-free AIN-93G pelleted diets were purchased from Dyets Inc. Bethlehem, PA, USA [13]. One control group and one deficient group (3 rats each) were killed at 3, 5, and 6 wk. The other rats were assigned to one of the following five treatments (6 rats per group) for an additional 2 or 4 wk.

- Free access to AIN-93G pelleted diet containing vitamin A at 4000 IU/kg and water (reference group).
- Gavage fed twice a week with 100 nmol/kgBW/d retinyl palmitate in cottonseed oil.
- Gavage fed twice a week with 100 nmol/kgBW/d retinyl palmitate provided as *β*-lactoglobulin complex in water. *β*-Lactoglobulin was prepared according to [14].
- Gavage fed twice a week with 100 nmol/kgBW/d retinyl palmitate carried by cottonseed oil in raw skim milk.
- Gavage fed twice a week with 100 nmol/kgBW/d retinyl palmitate provided as *β*-lactoglobulin complex in raw skim milk.

#### 2.1.2. Experiment 2

Weanling male Wistar rats were purchased from Charles River Laboratory (Raleigh, NC, USA). All rats were fed vitamins A and D deficient AIN-93G diet (Dyets, Inc., Bethlehem, PA, USA) upon arrival. Five rats were sacrificed within 3 days for the baseline data. All samples were stored at −20°C until analyses. Remaining rats were fed purified vitamin A and D deficient diet for 2 weeks and 5 rats were sacrificed for the serum and tissue analyses. The depletion continued for another 2 weeks, and another 5 rats were sacrificed for serum and tissue analyses. The rest of the rats were then randomly divided into the following five treatment groups.

- A/N, vitamin adequate diet (+Diet, regular AIN-93G pelleted rodent diet adequate in vitamin A) and nonfortified skim milk (NFM) as a replacement for drinking water (positive control group).
- D/N, vitamin deficient diet (−Diet, AIN-93G pelleted rodent diet without vitamin A or vitamin D) and nonfortified skim milk (NFM) as a replacement for drinking water (negative control group).
- D/P, vitamin deficient diet (−Diet) and protein-based vitamin fortified skim milk (PFM) as a replacement for drinking water.
- D/O, −Diet and oil-based vitamin fortified skim milk (OFM) as a replacement for drinking water.
- D/N/O, −Diet and a 2 : 1 mixture of NFM and OFM as a replacement for drinking water. Skim milk was fortified with 147 IU/100 mL of retinyl palmitate. The D/N/O treatment was added to the original experimental design to provide retinyl palmitate of equivalent amount to D/P, because the fortification level in PFM was about 1/3 of that in OFM, presumably due to unexpected thermal degradation in the UHT processing of PFM.

As their only water source, each day rats in all treatments described above were offered a fresh carton of UHT-processed skim milk in a glass water bottle. The milk consumed during the previous day was measured. Food consumption and rat weight gains were measured weekly. At 2 and 4 weeks after beginning the treatments, five rats from each group were sacrificed and serum and tissue samples were obtained. The research protocol was approved by the North Carolina State University Institutional Animal Care and Use Committee.

### 2.2. Materials

For experiment 1, *β*-Lactoglobulin was purified from acid whey by bioselective adsorption on N-retinyl-Celite, a technique that yields only nondenatured *β*-lactoglobulin devoid of lipid in the two binding sites [15]. For experiment 2, Biopure *β*-lactoglobulin was obtained from Davisco Foods International, Inc. (Eden Prairie, MN, USA). Fluorescence spectra were performed on a System 3 Scanning Spectrofluorometer (Optical Technology Devices, Inc., Elmsford, NY, USA) to verify binding of the *β*-lactoglobulin protein with vitamin A palmitate [16]. All chemicals, reagents, experimental materials, and dissection kits were obtained from either Sigma Chemical Co. (St. Louis, MO, USA) or Fisher Scientific (Pittsburgh, PA, USA) and were of highest analytical quality.

### 2.3. Gavage Preparations (Experiment 1)

Vitamin A-*β*-lactoglobulin complex stock was prepared in a 2 : 1 ratio of retinyl palmitate (dissolved in ethanol) and *β*-lactoglobulin (dissolved in distilled water), 0.26 mg of vitamin A in 1 mL of solution. The ethanol concentration in the fluids fed to the rats was lower than 3% [10]. The vitamin A-*β*-lactoglobulin complex solution (pH 6.41) was then divided into different vials and stored in the freezer for each gavage feeding preparation. Prior to each feeding, the vitamin A-*β*-lactoglobulin complex stock was homogenized with unfortified skim milk (pH 6.97 to final pH 6.81) or mixed with additional distilled water. Aliquots of vitamin A (retinyl palmitate) in cottonseed oil stock, 17.0 mg/mL, were prepared and stored in the freezer for individual gavage feeding treatment preparations. Unfortified skim milk was pasteurized in a 63°C water bath (Precision Scientific, Chicago, IL, USA) for 30 min. Prior to each feeding, the vitamin A oil stock was homogenized with unfortified skim milk or mixed with additional cottonseed oil.

### 2.4. Assays for Serum Retinol, Liver Retinol, and Retinyl Palmitate

#### 2.4.1. Serum and Liver Samples Preparation

In a disposable glass centrifuge tube, 200 *μ*L ethanol (0.01% BHT) was added into 200 *μ*L serum, and 0.1 *μ*g retinyl acetate in 100 *μ*L ethanol was added as the internal standard. Then 2 mL hexane was added, and the tubes were capped and vortexed vigorously for 1.5 min. After adding 100 *μ*L water, the tubes were vortexed for another 1.5 min. Then they were centrifuged for 5 min to separate phases. The upper hexane layer was transferred to amber sample vials for HPLC analyses [17]. One gram of liver homogenate was pipetted into a centrifuge tube wit 2 mL of 0.01% BHT ethanol, 50 *μ*L of 1 mg/mL retinyl acetate (internal standard), and 2 mL of hexane. The tube was capped and vortex-mixed vigorously for 1.5 min. Then uncapped and another 2 mL of hexane was added and vortex-mixed. A 500-*μ*L volume of distilled water was added and vortexed for 10 sec. The tube was centrifuged for 5 min. The upper hexane layer was transferred to an amber sample vial for HPLC analyses [18].

#### 2.4.2. HPLC Analyses for Liver and Serum Vitamin A

*Experiment  1.* Samples were analyzed by HPLC using Zorbax ODS, 7 mm, 4.6 × 150 mm silica column and 4.6 × 30 mm guard column (Phenomenex, Torrance, CA, USA). The mobile phase was HPLC grade pure methanol. Peaks from a SM 95 UVIS detector (Linear Instruments Co., Reno, NV, USA) set at 325 nm, were integrated with Dynamax HPLC Method Manager, version 1.2 (Rainin Instrument Co., Woburn, MA, USA). 

**Figure 1:**
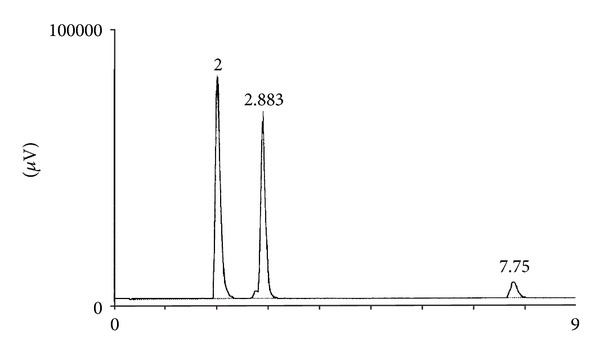
HPLC chromatogram of retinyl palmitate and retinol from the liver sample with retinyl acetate as internal standard: the retention times for retinyl palmitate, retinyl acetate, and retinol are 2.00, 2.88, and 7.75 min, respectively.

*Experiment  2.* HPLC analyses were performed on a Waters 510 pump system with UVIS 203 detector at a wavelength of 325 nm (*λ*_max⁡_) and U6K manual loading injector (Waters Associates, Milford, MA, USA). The 4.6 × 250 mm 5 *μ*m silica column and guard column (Prodigy, Phenomenex, Inc., Torrance, CA, USA) were used at room temperature. The isocratic mobile phase hexane/n-butyl chloride/acetonitrile (82 : 13 : 5, with 0.01 mL of acetic acid) was run at a flow rate of 1.5 mL/min [19]. A typical chromatogram of liver vitamin A is shown in Figure 1. The peak for retinyl palmitate was obtained in less than 8 min with this procedure, compared with nearly 30 min for the method used in experiment 1.

### 2.5. Statistical Analysis

*t*-test of two independent sample comparisons was used to determine the vitamin A deficiency condition. Data are expressed as means ± SD. Multiple sample completely randomized design analyses of variance were used to determine the overall effects of nutrient carriers, degree of vitamin A deficiency, and their interactions on the measured parameters. When an overall *F* ratio was significant (*P* < 0.05), group means were tested for significant difference at *P* < 0.05 with the Tukey HSD test.

## 3. Results

### 3.1. Experiment 1

#### 3.1.1. Growth Rate and Weight Comparison

Body weights and liver weights did not significantly differ between the vitamin-A-deficient group and the control group during deficiency development or during the first 2 wk of repletion (data not shown). However, there was a statistically significant difference in body weight and growth rate at 4 wk repletion (Table 1). Growth rate was significantly greater in nonmilk than in milk treatments. However, there was no significant difference between vitamin A carried by oil and vitamin A carried by *β*-lactoglobulin. The growth plateau is typical of vitamin A deficiency in rats [20].

#### 3.1.2. Serum Retinol Concentration

The effect of vitamin A deficiency on serum retinol concentration was detected as early as the 3rd wk after beginning experimental diets. Serum vitamin A concentrations were dramatically different at 6 wk deficiency, when serum vitamin A was 0.10 ± 0.023 mg/mL in deficient rats versus 0.684 ± 0.114 mg/mL in the regular diet group (Table 2).

**Figure 2:**
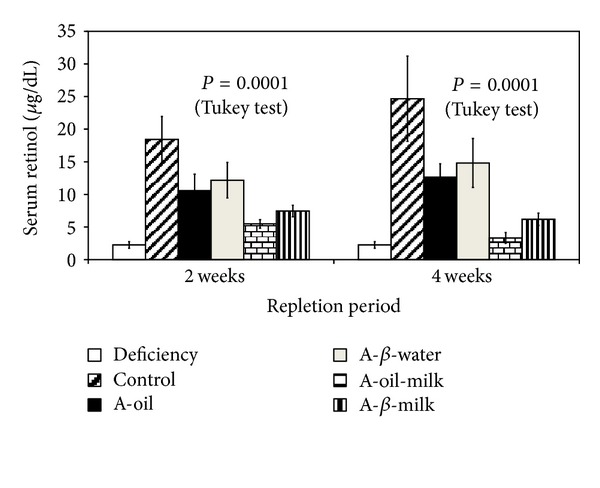
Serum retinol at 6 wk deficiency and 8 wk (2 wk repletion) and 10 wk (4 wk repletion). AIN-93G regular group is shown in the figure as reference information. Analysis of variance (ANOVA) of completely randomized multiple samples was used to compare 2 wk or 4 wk repletion between different gavage-fed treatments (not including the deficiency group). Mean values not sharing a letter are significantly different (*P* < 0.05). *n* = 6 rats/group.

Analysis of variance of completely randomized multiple samples was used to compare different gavage-fed treatment groups at 2 wk and 4 wk repletion. Serum retinol concentrations after 2 wk repletion and 4 wk repletion are shown in Figure 2. Serum vitamin A in the groups that received the *β*-lactoglobulin complexes was similar to that in groups that received vitamin A carried by oil either in water or skim milk. The results also showed significant differences in serum retinol concentrations between nonmilk and milk-containing treatment groups. At both 2 and 4 wk, the skim milk in the gavage-fed formulations significantly reduced the serum vitamin A relative to the respective nonmilk treatment (Figure 2).

#### 3.1.3. Liver Retinol and Retinyl Palmitate Concentration

The effect of vitamin A deficiency on liver vitamin A levels was detected as early as 3 wk after beginning experimental diets. Liver retinol and retinyl palmitate concentrations were dramatically different at 3, 4, and 6 wk of deficiency (Table 2). Liver retinol and retinyl palmitate concentrations after 2 and 4 wk repletion are shown in Table 3. Retinyl palmitate remained undetectable for this time in all treatments except “vitamin A in oil”.

### 3.2. Experiment 2

Due to relatively small liver vitamin A storage in rats, vitamin-deficient diet successfully depleted vitamin A in the rats during the 4-week depletion. The rats exhibited a normal progress of vitamin A deficiency: reduction of liver storage appeared first, followed by reduction in serum retinol.

Using the nutrient composition of AIN-93G, retinyl palmitate levels in skim milk, and food and milk consumption of rats, the actual vitamin A intake was calculated as shown in Table 4. After the 2-week repletion, no significant difference in serum retinol was detected among treatment groups, but liver retinol and retinyl palmitate levels were significantly higher in the groups A/N and D/O than the other groups (Table 5). After the 4 weeks repletion, serum retinol in the A/N group was significantly higher than the D/P group and serum retinol in the D/N group was significantly lower than all other groups. Liver vitamin A at 4 weeks of repletion exhibited a similar pattern to that after the 2 weeks of repletion (Table 6). During the repletion period, oil-based retinyl palmitate in skim milk repleted liver and serum vitamin A equivalent to vitamin-A-containing AIN-93G pelleted rodent diet, indicating similar bioavailability of retinyl palmitate in skim milk and pelleted diet. But no treatment had brought liver vitamin A status back to normal by the end of the repletion due to the limited amount of time in this phase of the study. As expected, no significant difference was detected in liver vitamin A between the D/N/O, D/P, and D/N groups, though the levels were higher in the first two groups. The D/N/O group was designed to provide the equivalent retinyl palmitate to PFM and the actual vitamin A intake between the two groups was similar (Table 4). Based on data from liver and serum vitamin A assays in these two groups, there was no significant difference in bioavailability of retinyl palmitate between PFM and OFM.

## 4. Discussion

Serum and liver vitamin A concentrations were similar to those reported [21] for depleted rats and those repleted with 5 *μ*g retinol/day, an amount similar to that used in the gavage-feeding study (100 nmol/kg BW/day). In the deficient rats, the repletion of serum vitamin A was slightly better (although not statistically significant) when rats were gavage-fed a vitamin A complex with *β*-lactoglobulin compared to when they were fed with an oil-based carrier (Figure 2). The two fortification methods for UHT milk also had similar effectiveness at repletion of serum retinol in experiment 2. Neither the gavage regimen (experiment 1) nor feeding UHT milk provided adequate vitamin A to significantly restore vitamin A during the experimental period.

Admixtures of TPN solutions which did not contain Intralipid for vitamin A solubilization showed reduction of vitamin A when stored in plastic bags; reduction was 35% after 48 hr at 5°C and 60% after 48 hr at 25°C [22]. Similar adsorption of vitamin A to milk plastic containers could cause a loss of vitamin A from fortified nonfat and skim milk packaged in polyethylene. Adsorption of vitamin A in skim milk to polyethylene has been demonstrated [23]. Because *β*-lactoglobulin is a water-soluble protein, it may prevent vitamin A, a lipid-soluble nutrient, from adsorbing to polyethylene packaging.

Vitamin A is unstable in the presence of light, oxygen, and oxidized fats and oils. Stability of skim milk fortified with all-trans-retinyl palmitate carried by various oil-based carriers was studied [24]. Greater loss occurred with corn oil as the vitamin carrier compared to coconut oil as carrier in various temperature and light conditions. Isomerization of all-trans to cis isomers of retinyl palmitate occurred in all oil-carrier systems [24]. Unfortunately, corn oil is often used as a carrier for oil-based vitamin A fortification in low-fat or skim milk, a practice that may not maximize vitamin A stability. This study used cottonseed oil as a comparison treatment to mimic the effects of a polyunsaturated oil as the solvent for vitamin A.

The hydrophobic binding capabilities of *β*-lactoglobulin have been studied by several researchers. Retinol and palmitate binding sites of *β*-lactoglobulin were reported in different publications [9, 10, 16, 25–29]. Retinyl palmitate may bind in both the *β*-barrel cavity and an external *α*-helix pocket. Wang and his colleagues [10] suggested that *β*-lactoglobulin was capable of binding 2 moles of retinyl palmitate per mol of protein. The hydrophobic binding activity of *β*-lactoglobulin might protect vitamin A from oxidation and isomerization. No previous reports were found to have compared bioavailability between fortified skim milk and nonmilk vitamin A sources in a rat model. *β*-Lactoglobulin addition to retinyl acetate-fortified whole bovine milk increased the serum vitamin A of calves more than did fortified milk without vitamin A. Our results from experiment 1 suggest that the skim milk reduces the bioavailability of both oil-carried and *β*-lactoglobulin-complexed forms compared to vitamin A given orally without milk.

**Figure 3:**
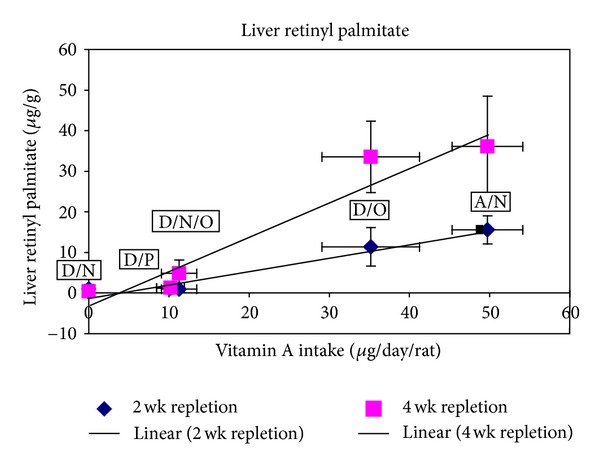
Linear regression of retinyl palmitate content of liver after 2-week or 4-week repletion with average vitamin A intake during Experiment 2. Points represent treatment groups ± standard deviation.

In experiment 2 the high-dose oil-fortified milk (**D/O**) adequately restored liver vitamin A, but the low doses did not. The regression of liver retinyl palmitate response to retinyl palmitate intake shows that the retinyl palmitate in the *β*-lactoglobulin complex was less effective at restoring liver retinyl palmitate than were other treatments (Figure 3). This finding could be consistent with the suggestion that retinol does not enter the liver as a RBP complex [30].

*β*-Lactoglobulin shows a high degree of homology in the amino acid sequence and conformational structure with human serum RBP. Transport of milk retinol in the intestine of newborns has been proposed as a role of *β*-lactoglobulin [9, 31], although others [12] speculate that this function is fortuitous, rather than an evolutionary design. Retinol was transported across a CaCo-2 cell monolayer with equal efficiency when it was complexed with *β*-lactoglobulin as when supplied as free retinol [32]. In contrast, *β*-lactoglobulin did promote uptake of vitamin D in a mouse model system [33].

The absorption of retinol-RBP might be receptor-mediated in the rat's intestinal brush border membranes, whereas the transport mechanism for free retinol is diffusion [34]. Because *β*-lactoglobulin is similar in structure to RBP, it might enhance the uptake of retinol [35]. *β*-Lactoglobulin is resistant to the proteolytic activity in the stomach and specific *β*-lactoglobulin receptors were found in intestinal cells [36], suggesting *β*-lactoglobulin may play a role in the delivery of retinol to the intestinal absorptive cells after hydrolysis of esterified retinol in the rumen. The effect of bovine milk *β*-lactoglobulin on intestinal uptake of retinol was examined in suckling rats with the everted gut-sac technique [35]. Uptake of retinol bound to *β*-lactoglobulin was significantly higher than that of free retinol both in the jejunum and the ileum. Perhaps if retinyl palmitate, the vitamin A form used in this experiment, is hydrolyzed by enzymes in the lumen, free retinol could follow the diffusion mechanism or rebind with *β*-lactoglobulin to be transported by a receptor mechanism into the intestinal cells. *β*-Lactoglobulin from milk might compete for receptors for retinol-*β*-lactoglobulin or retinol-RBP uptake and reduce the bioavailability of vitamin A added to milk. Further research should investigate this possibility and the use of these fortifiers in other nonfat foods and beverages.

## 5. Conclusion

The data reported here suggest that *β*-lactoglobulin can be used to complex vitamin A for fortification of milk and nonmilk liquid products with bioavailability similar to oil-based retinyl palmitate fortifier. Milk was an inhibitor of vitamin A utilization from either chemical form.

## Figures and Tables

**Table 1:** Effect of the supplementation vehicles on rat body, liver weight, and growth rate after 4 wk repletion of vitamin A^1^.

4 wk repletion of vitamin A treatments	Body weight (g)	Growth rate (g/day)	Liver weight (g)
Regular diet	449.67 ± 23.86^ab^	3.65 ± 0.31^ab^	10.98 ± 0.99
Vit A^2^ in oil	438.00 ± 5.57^abc^	3.73 ± 0.24^ab^	10.53 ± 0.28
Vit A^2^ in *β*-lact^2^	463.00 ± 5.20^a^	4.09 ± 0.57^a^	12.24 ± 0.78
Vit A^2 ^in Oil-milk	414.00 ± 10.54^c^	2.88 ± 0.25^c^	9.85 ± 0.79
Vit A^2^ in *β*-Lact^2^-milk	433.00 ± 15.59^bc^	3.42 ± 0.30^bc^	10.59 ± 1.11

^1^Values are Mean ± SD;  *n* = 3 rats/group. Means in a column with no common superscript letters are significantly different (*P* < 0.05).

^2^Vit A: vitamin A; *β*-Lact: -*β*-lactoglobulin.

**Table 2:** Serum and liver retinol and liver retinyl palmitate during vitamin A depletion (experiment 1)^1^.

	3 weeks	4 weeks	6 weeks
Serum	Retinol	(*μ*g/mL)	

Regular diet	0.498 ± 0.092	0.724 ± 0.263	0.682 ± 0.114
Deficient diet	0.409 ± 0.071	0.382 ± 0.098	0.100 ± 0.023

Liver	Retinol	(*μ*g/g)	

Regular diet	3.56 ± 0.83^a^	5.27 ± 1.05^a^	19.89 ± 5.78^a^
Deficient diet	1.17 ± 0.33^b^	1.20 ± 0.62^b^	0.12 ± 0.20^b∗^

Liver	Retinyl palmitate	(*μ*g/g)	

Regular diet	129.20 ± 17.36^A^	137.28 ± 25.26^A^	154.16 ± 45.31^A^
Deficient diet	20.40 ± 6.06^B^	9.37 ± 10.68^B^	0.00 ± 00.00^B∗∗^

^1^Values are mean ± SD; *n* = 3 rats/group. Means in a column for each compound with no common superscript letters are significantly different (*P* < 0.05).

*In one rat, vitamin A was not detectable by the HPLC analysis.

**No vitamin A was detectable by the HPLC analysis.

**Table 3:** Liver repletion retinol and retinyl palmitate after two and four weeks repletion (experiment 1)^1^.

	2-week repletion		4-week repletion	
	Retinol (*μ*g/g of liver)	Retinyl palmate (*μ*g/g of liver)	Retinol (*μ*g/g of liver)	Retinyl palmate (*μ*g/g of liver)
Regular	10.28 ± 0.98^a^	237.12 ± 24.93^ a^	18.79 ± 6.45^ a^	261.97 ± 26.52^ a^
Vit A in oil^2^	0.77 ± 0.07^b^	1.65 ± 0.57^ b^	1.89 ± 0.52^ b^	4.11 ± 2.34^ b^
Vit A in *β*-lact^2^	0.45 ± 0.02^ b^	0.00 ± 0.00^b∗^	0.63 ± 0.11^ b^	0.00 ± 0.00^b∗^
Vit A in oil-milk^2^	0.07 ± 0.04^ b^	0.00 ± 0.00^b∗^	0.00 ± 0.00^b∗^	0.00 ± 0.00^b∗^
Vit A in *β*-lact-milk^2^	0.44 ± 0.08^ b^	0.00 ± 0.00^b∗^	0.04 ± 0.07^b∗∗^	0.00 ± 0.00^b∗^

^1^Values are mean ± SD; *n* = 3 rats/group. Means in a column with no common superscript letters are significantly different (*P* < 0.05).

^2^Vit A: vitamin A; *β*-Lact: *β*-lactoglobulin.

*No vitamin A was detectable by the HPLC analysis.

**In one rat, vitamin A was not detectable by the HPLC analysis.

**Table 4:** Vitamin A intake (*µ*g/day/rat) as retinyl palmitate during repletion in experiment 2.

Time/group	A/N	D/P	D/O	D/N/O
Treatment description^1^	+Diet/NFM	−Diet/PFM	−Diet/OFM	−Diet/NFM : OFM

2-week repletion^2^	49.72 ± 3.08^a^	10.21 ± 0.63^c^	35.17 ± 5.33^b^	11.27 ± 1.66^c^
4-week repletion^2^	44.66 ± 4.40^a^	10.04 ± 1.73^b^	37.24 ± 6.09^a^	11.67 ± 2.22^b^

^1^+Diet: regular AIN-93G pelleted rodent diet adequate in vitamin A; −Diet: deficient AIN-93G pelleted rodent diet without vitamin A or vitamin D. One kg of the AIN-93 diet contains 4000 IU of all-trans retinyl palmitate. Skim milk was fortified with 147 IU/100 mL of retinyl palmitate.

^2^Each value is the mean ± SD. *N* = 4 in the A/N group. *N* = 5 in the other groups. Means in a row without common superscript letters are significantly different (*P* < 0.05).

**Table 5:** Liver and serum vitamin A after the 2-week repletion in experiment 2.

Parameters/group	A/N	D/N	D/P	D/O	D/N/O
Treatment description^1^	+Diet/NFM	−Diet/NFM	−Diet/PFM	−Diet/OFM	−Diet/NFM : OFM (2 : 1, v/v)

Serum retinol^2^ (*μ*g/mL)	0.40 ± 0.24	0.20 ± 0.08	0.29 ± 0.16	0.41 ± 0.23	0.30 ± 0.23
Liver retinol (*μ*g/g)	1.14 ± 0.09^a^	0^c^	0.01 ± 0.02^c^	0.79 ± 0.53^b^	0.15 ± 0.21^c^
Liver retinyl palmitate (*μ*g/g)	15.56 ± 3.46^a^	1.08 ± 0.46^b^	1.23 ± 0.80^b^	11.40 ± 4.76^a^	0.99 ± 0.69^b^

^1^See abbreviations.

^2^Data are shown as mean ± SD; *N* = 4 in the A/N group. *N* = 5 in the other groups. Means in a row without common superscript letters are significantly different (*P* < 0.05).

**Table 6:** Liver and serum vitamin A after the 4-week repletion in experiment 2.

Parameters/group	A/N	D/N	D/P	D/O	D/N/O
Treatment description^1^	+Diet/NFM	−Diet/NFM	−Diet/PFM	−Diet/OFM	−Diet/NFM : OFM (2 : 1, v/v)

Serum retinol^2 ^(*μ*g/mL)	0.57 ± 0.10^a^	0.10 ± 0.03^c^	0.34 ± 0.13^b^	0.38 ± 0.13^a,b^	0.46 ± 0.15^a,b^
Liver retinol (*μ*g/g)	1.53 ± 0.28^a^	0.11 ± 0.13^b^	0.30 ± 0.21^b^	0.84 ± 0.21^a^	0.39 ± 0.30^b^
Liver retinyl palmitate (*μ*g/g)	36.09 ± 12.39^a^	0.39 ± 0.15^b^	1.30 ± 0.55^b^	33.53 ± 8.79^a^	4.88 ± 3.23^b^

^1^See abbreviations.

^2^Each value is the mean ± SD and  *n* = 4 in negative and positive control groups (A/N and D/N), and  *n* = 5 in the other groups. Means in a row without common superscript letters are significantly different (*P* < 0.05).

## Abbreviations

RBP: Retinol-binding protein
AIN: American Institute of Nutrition
BW: Body weight
NFM: Nonfortified skim milk
PFM: Protein-based fortified skim milk
OFM: Oil-based fortified skim milk
UHT: Ultrahigh temperature
BHT: Butylated hydroxytoluene
HPLC: High pressure liquid chromatography
SD: Standard deviation
HSD: Highly significant difference
TPN: Total parenteral nutrition
+Diet: Regular AIN-93G pelleted rodent diet adequate in vitamin A
−Diet: Deficient AIN-93G pelleted rodent diet without vitamin A or vitamin D.
